# Stunting at birth: linear growth failure at an early age among newborns in Hawassa city public health hospitals, Sidama region, Ethiopia: a facility-based cross-sectional study

**DOI:** 10.1017/jns.2023.46

**Published:** 2023-05-30

**Authors:** Haileyesus Ejigu, Zelalem Tafese

**Affiliations:** 1 Sidama Public Health Institute as Regional Data Management Center for Health Coordinator, Hawassa, Ethiopia; 2 School of Nutrition, Food Science and Technology, Hawassa University, Hawassa, Ethiopia

**Keywords:** Ethiopia, Growth failure, Hawassa, Low birth weight, Newborn, Prevalence, Stunting

## Abstract

On a global basis, 144 million people are stunted, and in Ethiopia, it remains a major public health problem. A limited number of studies have been conducted at the national level and in the study area to generate information on stunting at birth. The present study investigated the magnitude and predictors of stunting among newborns delivered at the Public Hospitals of Hawassa City, Ethiopia. A facility-based cross-sectional study was conducted between August and September 2021 among mothers and newborns (*N* 371). Data were collected through face-to-face interviews with the mother in a waiting room after the delivery of the child at the hospital. Newborn length and weight were measured and converted to length-for-age *Z*-score using WHO standards. The prevalence of stunting at birth (35⋅6 %) and low birth weight (24⋅6 %) were high. In the adjusted model, factors significantly associated with stunting were birth interval <2 years, low birth weight, inadequate dietary diversity and food insecurity (*P* < 0⋅01) mid-upper arm circumference (MUAC) of mother <23 cm (*P* < 0⋅05). The high magnitude of stunting and low birth weight calls all stakeholders and nutrition actors to work on preventing maternal undernutrition and improving their dietary practice through nutrition education. It is also recommended to mitigate food insecurity with evidence-based interventions using a combination of measures. Additionally improving maternal health services including family spacing was recommended to reduce stunting and low birth weight among newborns in the study area.

## Introduction

Child undernutrition is a global problem with several concerns of survival, the incidence of acute and chronic diseases, healthy development and economic productivity of individuals and society^(1)^. Globally, more than one in four children under the age of 5 years are stunted and Sub-Saharan Africa and South Asia suffer the heaviest burden^(2)^. Stunting is one of the most common indicators of long-lasting undernutrition, which is a true growth failure or inability to reach potential height for a particular age^(3)^. World Health Organization (WHO) defined stunting as a height-for-age (HAZ) *Z*-score below 2 sd of the median of WHO standards^(4)^. It is also an indicator of chronic malnutrition resulting from a lack of adequate dietary intake over a long period and/or recurrent illness^(5)^. These primary causes of undernutrition are negatively affected by lack of food access, availability and healthcare services^(6)^. According to the estimates from UNICEF, the WHO and the World Bank, more than half of all stunted children <5 years old were reported to live in Asia, and more than one-third lived in Africa^(7)^. It is evidenced that Africa and Asia were regions highly affected by child stunting where they accounted for more than nine out of ten of all stunted children globally^(8)^.

In Ethiopia, stunting remains a major public health problem^(9)^. The same survey reported the national prevalence of stunting, among under-five children as 37 % and out of which 17⋅1 % were below 6 months of age. Other studies noted that nearly half of the children were stunted in the Northwest, Southern and Eastern parts of Ethiopia^(10–13)^.

The aetiology of stunting is varied but recognising the causal factors in the prenatal period including maternal height, weight gain, anaemia and infection, as well as the postnatal period such as infant and children's feeding practice, and infections becomes vital^(14–17)^. But the first 1000 days of life from conception, the age of 2 years is the most critical period in newborn growth^(18–20)^. It is less likely that stunted children regain the height lost as a result of stunting; consequently, most children will never gain the equivalent body weight for height^(21)^.

For stunting occurring at birth, maternal nutritional status plays a major role^(22)^. Prenatal causes of child stunting are directly associated with maternal undernutrition^(23)^. As a result, poor maternal nutrition during pregnancy can lead to a stunted newborn^(22–25)^; even though the effect of prenatal undernutrition may be addressed during the postnatal period through proper child-feeding practices^(25)^.

Safeguarding proper nutrition of pregnant women is an essential measurement to minimise stunting at birth^(20)^. Achieving so by helping women of reproductive age is in good nutritional status at conception is the best preventive measure^(20,22,23)^. Hence, stunting at birth is considered the overall best indicator of maternal nutritional, medical, obstetric and socioeconomic status^(21,26–28)^. It is also considered a simple and useful marker for assessing intergenerational linkages in health and malnutrition^(17)^.

In Ethiopia, even though the prevalence and associated factors of stunting among under-five children were addressed well, the nutritional status among newborns has been overlooked, while the burden is presumed to be high. Lots of efforts are focusing on mitigating undernutrition among children under 5 years age^(29–33)^. It is believed that evidence of stunting at birth is quite crucial to reaching the global target of reducing stunting by 40 % by 2025^(34)^, and also relevant to playing a role in the plan of the government of Ethiopia to end stunting^(35)^. Therefore, the primary aim of the present study was to assess the magnitude of stunting at birth and associated factors among those delivered at the Public Hospitals of Hawassa City. The magnitude of low birth weight and its coexistence with stunting was also investigated.

## Methods

### Study setting

A facility-based cross-sectional study was conducted between August and September 2021. The study was conducted among mothers who gave birth at Hawassa City Public Hospitals. Hawassa City is the capital of Sidama National Regional State, which is found 275 kilometres South of Addis Ababa. The City is subdivided into eight sub-cities of which seven are urban having 21 kebeles and one rural with 12 kebeles. According to the 2007 population census, the total number of people living in the city administration was 386 773 of which 195 320 are males and 191 453 are females. There are two public Hospitals in the city. Adare General Hospital is one of the Public Hospitals of Hawassa City established in 2015 G.C. and has an average monthly delivery flow of 490^(36)^. The other is Hawassa Referral Hospital which is a comprehensive specialised and teaching Hospital under Hawassa University. Presently, the Hospital has 400 total beds and an average monthly delivery flow of 540^(37)^.

### Sample size and sampling procedures

The sample size of 381 was calculated using the single population proportion formula with of 30⋅5 % prevalence of stunting at birth^(38)^, a 97 % confidence level and a 10 % non-response rate. The number of mother–child dyads to be selected was proportionally allocated to each Hospital according to the number of delivery flow reports of the same time interval of the preceding year. The *K*-value for each Hospital is distinctly calculated. Accordingly, the *K*-value for Hawassa Referral Hospital is *K* = 540/195 = 3, and for Adare General Hospital, *K* = 490/176 = 3. The first participant's mother was randomly selected by the lottery method. Since the *K*-value for both Hospitals is 3, every third newborn was selected using a simple random sampling technique until the required sample size is attained.

### Data collection

#### Socio-demographic characteristics

Data were collected through face-to-face interviews with the mother in a waiting room after the delivery of the child at the hospital. The socio-demographic variables were adapted from the Ethiopian Demographic Health Survey (EDHS) and others from similar previous studies. Basic socio-demographic data such as the age of the mother, marital status, estimated household's average monthly income, religion, educational status, occupation and data concerning household economic status were collected. Data collectors used interviewer-administered structured questionnaires to interview mothers by cross-checking their maternal medical cards when necessary.

#### Maternal feeding practices

The dietary diversity was calculated based on the number of food groups consumed by the mother women in the previous 24 h of the survey. We assigned a score of 1 to each food group if the subject had consumed it or 0 if not. The scores of the ten food groups were summed up to calculate the total dietary diversity score of women^(39)^.

#### Household Food Insecurity Access Scale

The Household Food Insecurity Access Scale (HFIAS)^(23)^ was used to assess household food insecurity during the 4 weeks preceding the survey. The households were categorised into four groups: food secure, mildly food insecure, moderately food insecure and severely food insecure. Finally, these were merged into two groups: food-secure and food-insecure households.

#### Anthropometric measurements

The anthropometric measurement was done immediately after birth. The weight of the newborns was measured by using a balanced digital Seca scale (Germany) to the nearest 100 g. The reading on each scale was taken to zero level before weighing each newborn.

The length of the newborn was measured when the newborn lay in a supine recumbent position. Two persons, one supported and secures the head of the newborn and the other took measurements of the newborn's length from the top of their head to the heel of their foot. The measurement was completed three times using an infantometer the average length of three measurements was recorded to the nearest 0⋅1 cm to ensure accuracy.

The height of the mothers was measured using a height board while the mother was in a standing position. Each height was taken to the nearest 1 cm. The MUAC of the mother was measured with non-stretchable standard tape to the nearest 0⋅1 cm.

#### Data processing and analysis

Data were checked, coded and entered into SPSS, v. 20, for analysis. HAZ was calculated using the WHO Child Growth Standards^(4)^. WHO Anthro software was used to convert the anthropometric measurements into WHO *Z*-scores. Stunting was defined as HAZ <−2⋅0 and categorised using WHO definitions. Low birth weight was determined according to WHO standard birth weight below 2500 g^(40)^.

Descriptive statistics, including percentages as well as means and standard deviation (sd), were used to describe the characteristics of the study population. The normal distribution of the data was checked with the Kolmogorov–Smirnov test^(41)^.

Bivariate logistic regression analysis with crude odds ratio at 95 %CI was used to assess the association between dependent and independent variables. Multivariate logistic regression analysis with an adjusted odds ratio at 95 %CI was conducted to determine predictors of undernutrition and associations were declared significant at *P* ≤ 0⋅05. Multi-collinearity was checked among independent variables, the variance inflation factor (VIF) was found to be less than 1⋅12 and the tolerance test was found to be 0⋅89. The final model fitness status was checked by Hosmer and Lemeshow's goodness-of-fit chi-square test (*P*-value = 0⋅391).

## Results

### Socio-demographic characteristics

Among the 381 recruited mother–newborns pairs, 362 participated in the study giving a response rate of 95 %. Forty-six (12⋅7 %) of the study mothers were in the age group of <20 years old, while 284 (78⋅5 %) mothers were in the 20–34 years range and 13 (3⋅6 %) had no formal education (Table 1).

**Table 1.** Socio-demographic characteristics of participant mothers (*N* 362), at Public Hospitals, Hawassa City, Ethiopia, August–September 2021

Variables		Percentage (%) or mean (sd)
Mother age at birth (year)	<20	12⋅7
	20–34	78⋅5
	≥35	8⋅8
Mothers level of education	No formal education	3⋅6
	Primary school	11⋅9
	Secondary school	54⋅7
	College and above	29⋅8
Father level of education	No formal education	3⋅6
	Primary school	11⋅9
	Secondary school	55⋅5
	College and above	29⋅0
Mother occupation	House wife	11⋅9
	Merchant	14⋅9
	Public service employee	71⋅0
	Others*	2⋅2
Father occupation	Daily labourer	12⋅4
	Merchant	15⋅2
	Public employee	70⋅4
	Others (student, farmers)	1⋅9
Monthly family income (Eth.Birr)**	<2000	25⋅1
	2000–5000	52⋅8
	≥5000	22⋅1

* Others: students and daily labourer.

** 1 US$ = 42 Eth.Birr.

### Obstetric and maternal health characteristics

The majority of 300 (82⋅9 %) mothers was multipara and only 50 (13⋅8 %) was primipara. Two hundred seventy-six (76⋅2 %) had a birth interval of ≥24 months. About 323 (89⋅2 %) had antenatal care (ANC) during pregnancy, and 234 (71⋅3 %) had taken iron supplementation during their recent pregnancy. About 59 (16⋅3 %) women had pregnancy-induced hypertension (PIH), while 77 (21⋅3 %) women gave birth with gestational age <37 weeks (Table 2).

**Table 2.** Health, nutritional and life behavioural characteristics of mothers (*N* 362), at Public Hospitals, Hawassa City, Ethiopia, August–September 2021

Variables		Percent%
Parity	Multipara^a^	82⋅9
	Primpara^b^	17⋅1
Birth interval	≥24 months	76⋅2
	<24 months	23⋅8
Number of antenatal care visit (ANC)	≥4 times	34⋅3
	<4 times	65⋅7
Had medically confirmed diseases^c^		12⋅7
History of pregnancy-induced hypertension (PIH)		
Unintended pregnancy		9⋅1
Gestational age the child was born	≥37 weeks	78⋅7
	<37 weeks	21⋅3
Had history of smoking		11⋅6
Had history of Khat chewing^d^		6⋅6
MUAC	≥23 cm	79⋅3
	<23 cm	20⋅7
Mothers who had iron supplements during pregnancy	No	28⋅7
	Yes	71⋅3
Maternal height	≥150 cm	88⋅7
	<150 cm	11⋅3
Meal frequency of the mother	≥4 times	78⋅7
	<4 times	21⋅3
Dietary diversity of the mother	Adequate	77⋅6
	Inadequate	22⋅4
Food security status	Secure	41⋅4
	Insecure	58⋅6

a Primipara: A woman who gave birth one time.

b Multipara: A woman gave more than one birth.

c Heart disease, kidney disease, malignancy.

d A leaf chewed for its stimulant effect.

### Nutritional characteristics of mothers

Two hundred eighty-seven (79⋅3 %) mothers’ MUAC is measured at ≥23 cm. About 281 (77⋅6 %) mothers had adequate dietary diversity, and 212 (58⋅6 %) mothers were food insecure (Table 2).

### Newborn anthropometric characteristics

**Fig. 1.**
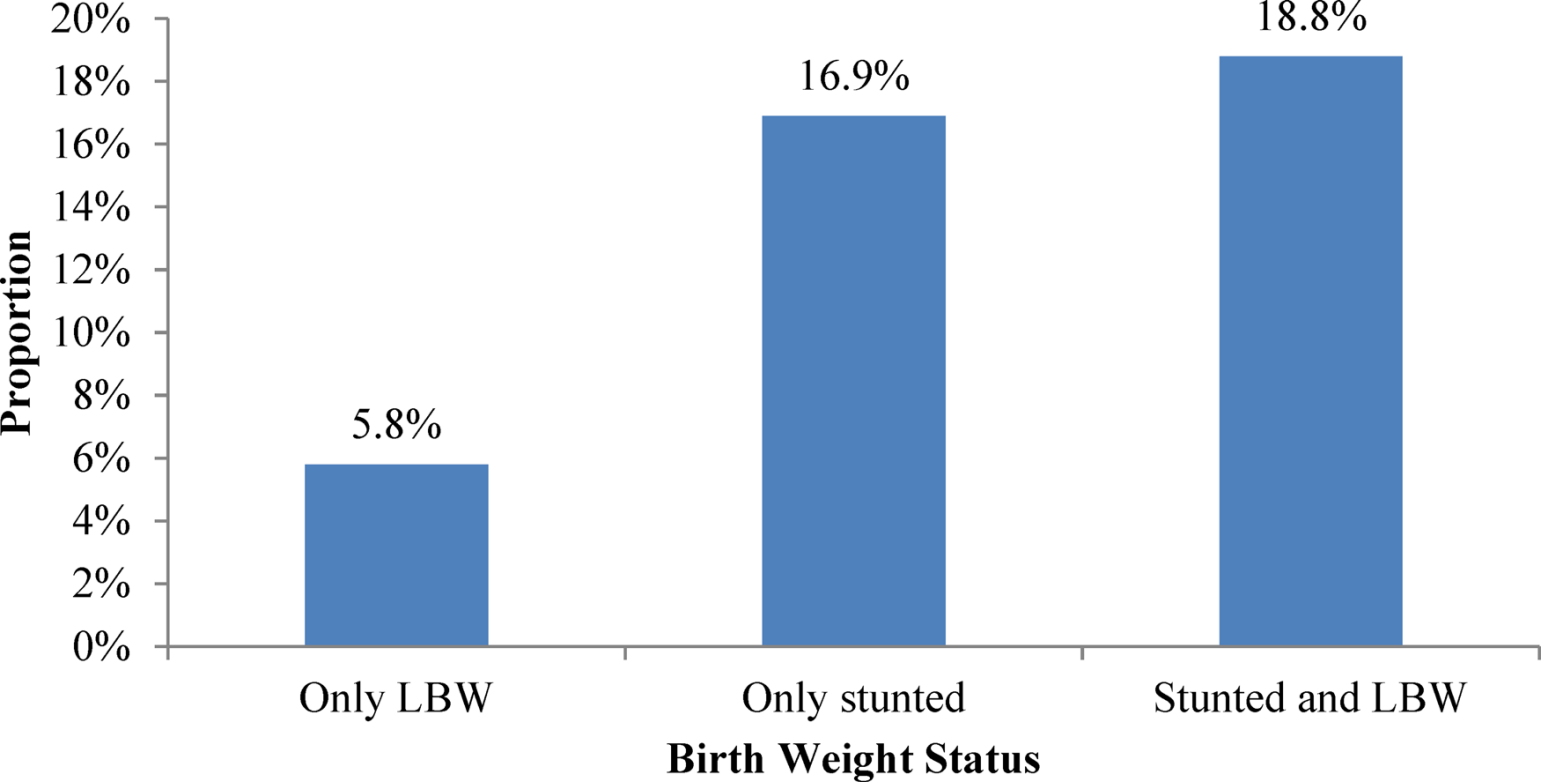
Low birth weight and stunting among newborns (*N* 362) delivered at Public Hospitals, Hawassa City, Ethiopia, August–September 2021.

One hundred twenty-nine (35⋅6 %) newborns had a length-for-age *Z*-score below −2 sd, and 89 (24⋅6 %) newborns were low birth weight. Twenty-one (5⋅8 %), 61 (16⋅9 %) and 68 (18⋅8 %) newborns were only low birth weight, only stunted and stunted and low birth weight, respectively (Fig. 1).

### Factors associated with stunting at birth

In a bivariate analysis, birth interval of <24 months (*P* < 0⋅001), age of mother <20 years (*P* < 0⋅05), not getting iron supplements during pregnancy (*P* = 0⋅004), presence of PIH (*P* = 0⋅141), giving birth before <37 weeks (*P* < 0⋅05), mothers MUAC <23 cm (*P* < 0⋅01), inadequate dietary diversity, being in food-insecure household (*P* < 0⋅01) and low birth weight (*P* < 0⋅01) predicted stunting at birth (Table 3). However, in a multivariate logistic regression model that adjusted for covariates (Table 3), low birth weight, being in a food-insecure household, inadequate dietary diversity of mother, birth interval of <24 months and mothers MUAC <23 cm remained statistically significant. The AOR of low birth weight with stunting at birth was 10⋅9 (AOR 10⋅9; 95 % CI 5⋅85, 20⋅30), being in food-insecure households 2⋅56 (AOR 2⋅56; 95 % CI 1⋅46, 4⋅49) and inadequate diet diversity had >4 times higher likelihood of giving stunted newborn 4⋅03 (AOR 4⋅03; 95 % CI 2⋅18, 7⋅48). Likewise, a 2⋅13 times higher likelihood of stunting at birth was found among newborns from mothers with MUAC <23 cm (AOR 2⋅13; 95 % CI 1⋅13, 4⋅01), compared with a newborn from mothers with MUAC ≥ 23 cm. Our model also detected a more than double likelihood of newborn stunting among mothers with a birth interval of <24 months (AOR 2⋅55; 95 % CI 1⋅39, 4⋅69), compared to those mothers with a birth interval of ≥24 months (Table 3).

**Table 3.** Factors predicting the likelihood of stunting at birth (*N* 362), at Public Hospitals, Hawassa City, Ethiopia, August–September 2021

Variables		COR (95 % CI)	*P*-value	AOR (95 % CI)	*P*-value*
Age of the mother	<20 years	3⋅32 (1⋅26, 8⋅73)	0⋅015	1⋅16 (0⋅42, 3⋅15)	0⋅05
	20–34 years	1⋅26 (0⋅56, 2⋅84)	0⋅570	1⋅95 (0⋅59, 6⋅45)	
	≥35 years^a^	1		1	
Birth interval	<24 months	2⋅67 (1⋅63, 7⋅53)	0⋅001	2⋅55 (1⋅39, 4⋅69)	0⋅001
	≥24 months^a^	1		1	
Gestational age	≥37 weeks^a^	1		1	
	<37 weeks	1⋅69 (1⋅04, 2⋅83)	0⋅044	1⋅69 (0⋅90, 3⋅17)	
Birth weight	Low	11⋅25 (6⋅39, 19⋅82)	0⋅001	10⋅9 (5⋅85, 20⋅30)	<0⋅01
	Normal^a^	1		1	
MUAC	≥23 cm^a^	1		1	
	<23 cm	2⋅54 (1⋅52, 4⋅27)	0⋅001	2⋅13 (1⋅13, 4⋅01)	0⋅018
Dietary diversity	Adequate^a^	1		1	
	Inadequate	3⋅59 (2⋅15, 5⋅00)	0⋅001	4⋅03 (2⋅18, 7⋅48)	<0⋅01
Food security	Secure^a^	1		1	
	Insecure	2⋅22 (1⋅40, 3⋅50)	0⋅001	2⋅56 (1⋅46, 4⋅49)	0⋅01
Mother had iron supplement during pregnancy	No	2⋅11 (1⋅29, 3⋅45)	0⋅003	1⋅84 (0⋅99, 3⋅41)	
	Yes		1		

AOR, adjusted odds ratio; COR: crude odds ratio; MUAC, mid-upper arm circumference.

a Reference categories.

* Statistically significant at *P* < 0⋅05.

## Discussion

The high prevalence of stunting at birth 35⋅6 % (30⋅7–40⋅6 %) among newborns in the present study suggests severe public health significance of problems needing evidence-based intervention. The findings on the prevalence of stunting at birth are consistent with previous studies from Guatemala (33 %)^(20)^, but much higher than stunting rates reported in Indonesia (10⋅2 %) and Ethiopia (30⋅5 %)^(42,38)^. Even though an association between gestational age and the short stature of mothers with stunting at birth has been reported in the previous study^(42)^, our study did not show significant associations of this variable. In the present study, birth interval between two consecutive births had a statistically significant association with stunting at birth. According to WHO technical consultation group on birth spacing after having live birth, the recommended interval before attempting the next pregnancy is at least 24 months which can reduce the risk of adverse maternal, perinatal and infant outcomes^(43)^. Consistently, the present study revealed an increased likelihood of stunting at birth among newborns delivered with a birth interval of <24 months compared with those delivered with a birth interval ≥24 months. Unlike the findings of previous studies^(42,44)^, our findings did not show a significant association between being delivered from a short-stature mother as a determinant of stunting at birth. Similarly, there was a lack of significant association between stunting with not having ANC in the present study but reported in previous studies to have a significant association for children born from mothers who did not have ANC^(13,45,46)^. Possibly this might be due to the majority of mothers in the present study being multipara and they may get adequate nutrition information during their previous pregnancies. Additionally, the present study was conducted in an urban area for the study participants to access mass media and get adequate health and nutrition information.

Consistent with the findings of previous studies^(47–50)^, the present study revealed a significant association between maternal dietary diversity and food insecurity in newborn stunting. The possible explanation might be food access and diversification during pregnancy enable to nourishing of the fetus for better growth and development during fetal life. This is because an inadequately diversified diet lacks some essential nutrients which would be essential for fetal growth and development consequently leading to newborn growth failure.

Inline, the present study indicated that the likelihood of stunting was increased for newborns delivered from mothers with poor nutritional status (MUAC < 23 cm) compared with their counterparts. This may be related to a lack of access to a nutritional diet, knowledge deficit of good maternal dietary practice or mistaken perception of women that frequent and much diet consumption during pregnancy could lead to excessive fetal growth which they perceive would be beyond tolerance of the birth canal and result with difficulty during childbirth^(26,51–53)^. Due to this, pregnant women might not eat adequately, thus, being exposed to undernutrition and can give stunted newborns^(54)^.

Low birth weight is one of the crucial factors in our model which had a significant association with newborn stunting, and a similar finding was reported in previous studies^(44,53–55)^. The plausible reason behind this might be low birth weight is an indicator of premature birth or intrauterine growth restriction, and hence, low birth weight is a predisposing factor to child growth failure resulting in stunted birth^(56)^.

## Conclusion

The present study has limitations that need to be considered including the cross-sectional nature of the study does not allow causal inferences to be made. A certain level of recall bias should be taken into consideration concerning the last menstrual period, dietary practice and medical history of the mother.

Additionally, the causal factors behind the high proportion of stunting at birth are not thoroughly addressed from intergenerational malnutrition perspectives. Future studies can help understand the causal factors leading to stunting at birth and help design effective interventions to reduce the problem. However, the present study is one of a few studies in Ethiopia and elsewhere that assess the prevalence and predictors of stunting at birth.

Despite the above limitations, our data provide important information on the prevalence and potential predictors of stunting which is important for policy makers and funders. Our data also highlighted the prevalence of stunting at birth and low birth weight is high, needing critical attention by all nutrition actors. Newborns delivered with low birth weight are more stunted. Similarly, stunting is widely witnessed among newborns delivered with a birth interval between two consecutive births <24 months, undernourished mothers, inadequate dietary diversity of women during pregnancy and household food insecurity. Preventing maternal undernutrition through nutrition education is emphasised. It is also crucial to give due emphasis to improving maternal health services including family planning and interventions to minimise low birth by providing all necessary services for all pregnant women during pregnancy.

It is also recommended to strengthen the existing community-level interventions for improving access to food, particularly for the most vulnerable groups of the community, women during pregnancy and lactation. The present study is conducted in only Public Hospitals; women who gave birth in private clinics/Hospitals were not considered. Consequently, this may underestimate or overestimate the rates. The estimate might be better represented if it includes all health institutions, and longitudinal follow-up data were used.
